# Calmodulin promotes matrix metalloproteinase 9 production and cell migration by inhibiting the ubiquitination and degradation of TBC1D3 oncoprotein in human breast cancer cells

**DOI:** 10.18632/oncotarget.16756

**Published:** 2017-03-31

**Authors:** Huzi Zhao, Lina Zhang, Yongchen Zhang, Lei Zhao, Qing Wan, Bei Wang, Xiaodong Bu, Meiling Wan, Chuanlu Shen

**Affiliations:** ^1^ Department of Pathology and Pathophysiology, Medical School, Southeast University, Nanjing, Jiangsu, People's Republic of China

**Keywords:** TBC1D3, calmodulin, protein degradation, protein ubiquitination, cell migration

## Abstract

The hominoid oncoprotein TBC1D3 enhances growth factor (GF) signaling and GF signaling, conversely, induces the ubiquitination and subsequent degradation of TBC1D3. However, little is known regarding the regulation of this degradation, and the role of TBC1D3 in the progression of tumors has also not been defined. In the present study, we demonstrated that calmodulin (CaM), a ubiquitous cellular calcium sensor, specifically interacted with TBC1D3 in a Ca^2+^-dependent manner and inhibited GF signaling-induced ubiquitination and degradation of the oncoprotein in both cytoplasm and nucleus of human breast cancer cells. The CaM-interacting site of TBC1D3 was mapped to amino acids 157~171, which comprises two 1–14 hydrophobic motifs and one lysine residue (K166). Deletion of these motifs was shown to abolish interaction between TBC1D3 and CaM. Surprisingly, this deletion mutation caused inability of GF signaling to induce the ubiquitination and subsequent degradation of TBC1D3. In agreement with this, we identified lysine residue 166 within the CaM-interacting motifs of TBC1D3 as the actual site for the GF signaling-induced ubiquitination using mutational analysis. Point mutation of this lysine residue exhibited the same effect on TBC1D3 as the deletion mutant, suggesting that CaM inhibits GF signaling-induced degradation of TBC1D3 by occluding its ubiquitination at K166. Notably, we found that TBC1D3 promoted the expression and activation of MMP-9 and the migration of MCF-7 cells. Furthermore, interaction with CaM considerably enhanced such effect of TBC1D3. Taken together, our work reveals a novel model by which CaM promotes cell migration through inhibiting the ubiquitination and degradation of TBC1D3.

## INTRODUCTION

Intracellular protein degradation is a vital process that is required for cells to recognize and eliminate damaged protein species and to control protein abundance in accordance with functional need. This process plays a critical role in diverse fundamental cellular functions such as cell cycle, growth, differentiation and cell migration. Aberrations in protein degradation have been implicated in the pathogenesis of numerous diseases, especially neurodegenerative, inflammatory and malignant diseases [1–3].

The intracellular degradation of proteins is mainly accomplished in two ways: the autophagy-lysosomal protein degradation machinery and the ubiquitin-proteasome system (UPS) [4]. The protein degradation performed through UPS involves ubiquitin, three classes of enzymes required for protein ubiquitination, and proteasome. A substrate protein will be degraded by the 26S proteasome, the multi-protein protease present in both cytoplasm and the nucleus once polyubiquitination occurs through a cascade reaction requiring the successive action of three enzymes including ubiquitin-activating enzyme (E1), ubiquitin-conjugating enzyme (E2) and ubiquitin ligase (E3). There are more than 1000 E3 ligases in human, which provide the specificity required for selective protein degradation [5, 6]. The E3 enzymes are divided into four classes on the basis of their biochemical and structural features: HECT, RING-finger, U-box, and PHD-finger types [7]. The RING-finger E3 ligases are the largest class and further fall into subfamilies, including cullin-based E3 enzymes such as Skp1–Cullin–F-box-protein (SCF) complexes [8]. SCF complexes consist of four proteins: Rbx1 (RING-box protein 1), Cullin, Skp1 (S-phase kinase associated protein 1) and F-box protein. The human genome encodes eight cullin proteins (CUL1 to CUL7 and PARC) and about 70 F-box proteins, which contain a cullin homology domain and at least one F-box domain, respectively [9, 10]. The F-box protein subunit recognizes and binds to most SCF substrates only when post-translational modifications (PTMs) including phosphorylation occur to them [11]. PTMs such as phosphorylation and acetylation have been shown to regulate the UPS-dependent degradation of both metastasis-suppressing protein RAPGEF2 and metastasis-promoting proteins including SNAIL, Twist1, HSPA5 and HIF-1α in breast cancer cells [12–17].

Besides PTMs, the UPS-dependent degradation of proteins has been subjected to the regulation by calmodulin (CaM), a ubiquitous and evolutionarily conserved Ca^2+^-binding protein [18]. CaM interacts with multiple target proteins in a Ca^2+^-dependent or independent manner [18]. One of the target proteins is estrogen receptor-α (ER-α), which is an estrogen-activated transcription factor and participates in metabolism, cell growth and development as well as tumorigenesis [19]. CaM is often overexpressed in breast cancers, especially in ER-positive breast tumors and inhibits the degradation of ER-α [20, 21].

Our laboratory has focused on the regulation of TBC1D3 degradation as well as its role in tumor development and progression. *TBC1D3* (also referred to as prostate cancer gene 17, PRC17) was identified as a hominoid-specific gene, with only one copy in the chimp genome and 5 ~ 53 copies in the human genome depending on ethnic origin [22–24]. This gene is widely expressed in human tissues and overexpressed in prostate, breast, bladder and pancreatic cancer as well as in myelodysplastic syndrome (MDS) [22, 25–28]. Ectopic expression of *TBC1D3* confers tumorigenicity to mouse NIH 3T3 embryonic fibroblast cells, indicating that *TBC1D3* functions as an oncogene [25]. Structurally, the *TBC1D3* oncogene belongs to the superfamily of human TBC-containing genes, with the TBC (Tre-2/Bub2/Cdc16) domain generally encoding GTPase-activating proteins (GAPs) for Rab family GTPases [29]. However, TBC1D3 protein has no GAP activity owing to the absence of the conserved arginine and glutamine residues required for the catalytic activity of the TBC domain [30]. Rather, TBC1D3 inhibits the ubiquitination of epidermal growth factor receptor (EGFR) and insulin receptor substrate-1 (IRS-1) by c-Cbl and Skp1-CUL7-Fbxw8 (SCF-FBXW8) E3 ubiquitin ligases, respectively, and their subsequent degradation, thereby enhancing EGF and insulin signaling and consequential cell proliferation [31, 32]. Our recent work identified TBC1D3 as a novel nucleocytoplasmic protein, cytoplasmic retention of which by microtubule network is required for the enhanced EGF signaling [33]. Conversely, growth factor (GF) signaling promotes SCF-FBXW8 E3 ubiquitin ligases-mediated TBC1D3 ubiquitination and proteasomal degradation, which is suppressed by TBC1D3 palmitoylation, another PTM [34, 35]. However, aside from these studies, little else is known of how the ubiquitination and degradation of TBC1D3 are regulated. Furthermore, the role of TBC1D3 in aggressive tumor behavior remains completely undefined.

In the present study, we demonstrate that CaM specifically interacts with TBC1D3 in a Ca^2+^-dependent manner and inhibits GF signaling-induced ubiquitination and degradation of the oncoprotein in both cytoplasm and the nucleus of human breast cancer cells. We also identify lysine residue 166 within the CaM-interacting motifs of TBC1D3 as the actual site for the ubiquitination. Point mutation of this lysine residue causes inability of GF signaling to induce the ubiquitination and subsequent degradation of TBC1D3. Finally, we find that TBC1D3 promotes the expression and activation of MMP-9 and the migration of human breast cancer cells, and interaction with CaM considerably enhances such effect of TBC1D3. Our work thus reveals a novel mode by which CaM promotes cell migration through inhibiting the ubiquitination and degradation of TBC1D3.

## RESULTS

### Calmodulin inhibits the FCS-induced ubiquitination and degradation of TBC1D3 in both cytoplasm and the nucleus

**Figure 1:**
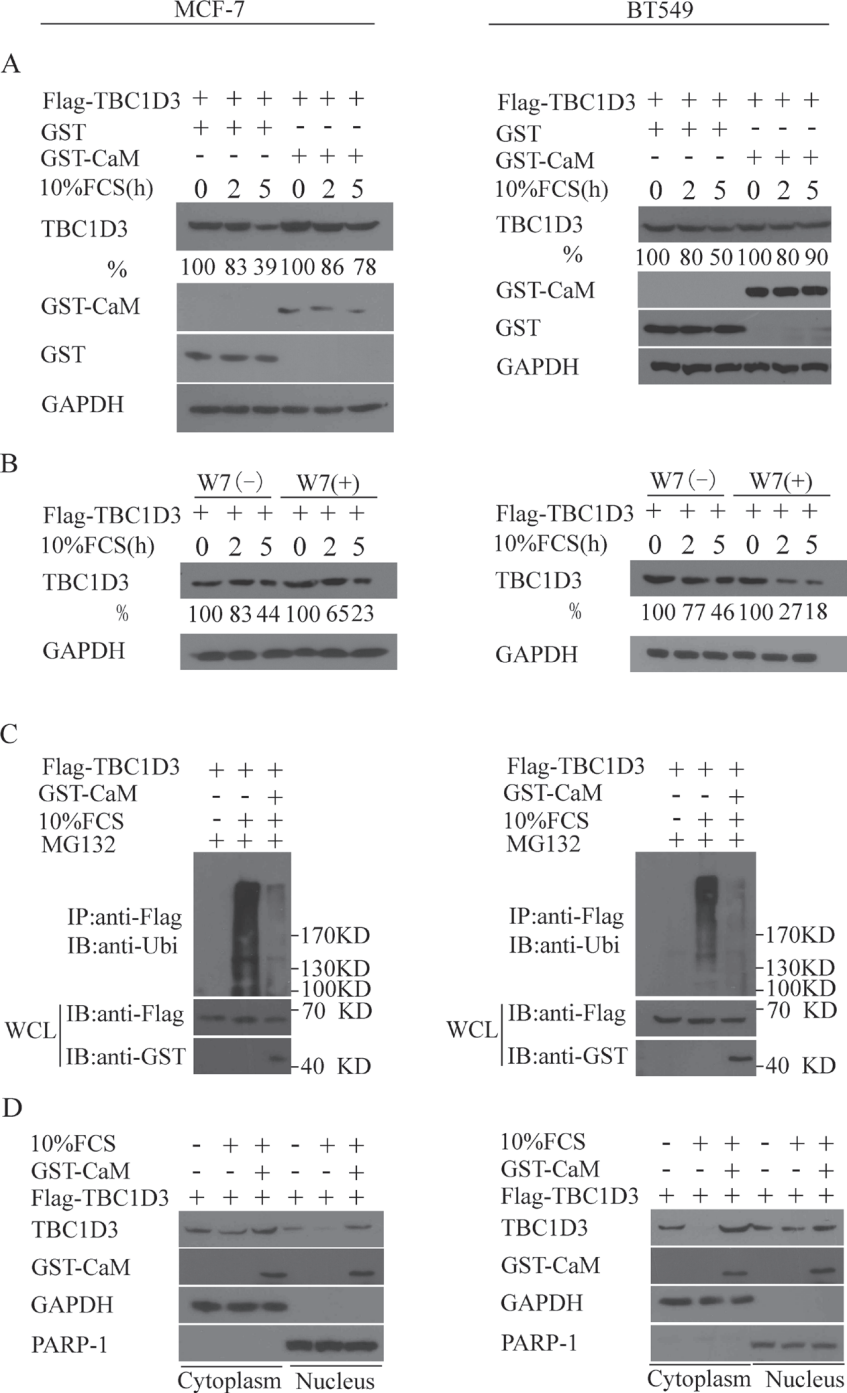
CaM inhibits the ubiquitination and degradation of TBC1D3 in both cytoplasm and the nucleus in response to FCS stimulation (**A**–**B**) MCF-7 and BT549 cells (left and right panels, respectively) were transfected with Flag-TBC1D3 alone (B) or together with GST-CaM or control GST vector (A). After 20 h, the cells were starved in serum-free medium with or without W7 (90 μM) for 3 h, and then stimulated by the addition of 10% fetal calf serum (FCS) in the presence of cycloheximide (25 μg/ml) for the indicated time. Cell extracts were resolved by SDS-PAGE and immunoblotted with anti-Flag (TBC1D3), anti-GST and anti-GAPDH antibodies. The number below each blot represents the percent TBC1D3 remaining at the indicated time point of FCS treatment normalized to the zero time point. (**C**) MCF-7 and BT549 cells (left and right panels, respectively) were transfected with Flag-TBC1D3 alone or together with GST-CaM. After 20 h, the cells were serum-starved in the presence of MG132 for 6 h, and then stimulated with (+) or without (−) 10% FCS for 20 min. Lysates were immunoprecipitated (IP) with anti-Flag antibody. After SDS-PAGE, Flag-TBC1D3, GST-CaM and polyubiquitinated TBC1D3 were immunoblotted (IB) with anti-Flag, anti-GST and anti-ubiquitin (Ubi) antibodies, respectively. Molecular weight markers are shown on right in kD. WCL, whole cell lysate. (**D**) MCF-7 and BT549 cells (left and right panels, respectively) were transfected with Flag-TBC1D3 alone or together with GST-CaM. After 20 h, the cells were starved in serum-free medium for 3 h, stimulated with (+) or without (−) 10% fetal calf serum (FCS) in the presence of cycloheximide (25 μg/ml) for 3 h, and then subjected to cell fractionation. Cytoplasmic and nucleic extracts were resolved by SDS-PAGE and immunoblotted with anti-Flag, anti-GST, anti-PARP-1 and anti-GAPDH antibodies.

Since calmodulin (CaM), a ubiquitous cellular calcium sensor, is often overexpressed in breast cancers, especially in estrogen receptor-positive breast tumors and enhances the stability of estrogen receptor [20, 21], we examined whether it also protects TBC1D3 from GF-induced degradation in two distinct cell culture models of human breast cancer, MCF-7 and BT549 cell lines. MCF-7 and BT549 are estrogen receptor-positive and -negative breast cancer cells, respectively [36, 37]. As shown in Figure 1A (left panel), MCF-7 cells transfected with GST vector showed a substantial degradation of TBC1D3; after 2 hours of fetal calf serum (FCS) stimulation, approximate 20% of TBC1D3 proteins were lost, and less than 40% of these proteins were left after 5 hours. In contrast, TBC1D3 degradation was significantly delayed in cells overexpressing CaM; less than 15% of TBC1D3 proteins were degraded after 2 hours, and about 80% of TBC1D3 proteins persisted after 5 hours (left panel in Figure 1A). Similarly, CaM overexpression substantially increased the stability of TBC1D3 in BT549 cells in response to FCS stimulation (right panel in Figure 1A). These results suggest that CaM overexpression inhibits the FCS-induced degradation of TBC1D3 in both estrogen receptor-positive and -negative human breast cancer cells.

We next asked whether endogenous CaM protects TBC1D3 from GF-induced degradation in human breast cancer cells. To address this issue, we performed similar experimens with W7, a calmodulin antagonist. Treatment of MCF-7 and BT549 cells (left and right panels in Figure 1B, respectively) with W7 substantially increased the FCS-induced degradation of TBC1D3. These results suggest that endogenous CaM suppresses the degradation of TBC1D3 in human breast cancer cells in response to FCS stimulation.

Since polyubiquitination plays a vital role in the degradation of TBC1D3 in response to GF signaling [34], we examined the ubiquitination of TBC1D3 in MCF-7 and BT549 cells transfected with Flag-TBC1D3 alone or together with GST-CaM. As seen in Figure 1C, when the degradation of TBC1D3 was blocked by a proteasome inhibitor MG132, polyubiquitinated TBC1D3 substantially existed in MCF-7 (left panel) and BT549 (right panel) cells transfected with Flag-TBC1D3 alone in the presence of FCS stimulation. However, CaM overexpression drastically reduced the FCS-induced ubiquitination of TBC1D3 (Figure 1C), further supporting a role for CaM in suppressing the FCS-induced degradation of TBC1D3 in human breast cancer cells.

The UPS-mediated protein degradation occurs in both the cytoplasm and the nucleus [38]. Recently, we identified TBC1D3 as a nucleocytoplasmic protein, and its association with tubulin/microtubule regulates its nucleocytoplasmic distribution [33]. However, it was unknown where TBC1D3 is degraded through the UPS and where CaM inhibits the FCS-induced degradation of TBC1D3. To address these issues, we prepared cytoplasmic and nuclear extracts from MCF-7 and BT549 cells (left and right panels in Figure 1D, respectively) transfected with Flag-TBC1D3 alone or together with GST-CaM and examined the FCS-induced degradation of TBC1D3 in the presence or absence of CaM overexpression. The FCS stimulation resulted in TBC1D3 degradation in both cytoplasm and the nucleus (Figure 1D). In contrast, overexpression of CaM abolished such effect of FCS on TBC1D3 stability in the above two subcellular localizations (Figure 1D). Consistent with these results, CaM had no impact on the localization of TBC1D3 in MCF-7 cells (Supplementary Figure 1). Together, these data reveal that CaM inhibits the GF-induced ubiquitination and degradation of TBC1D3 in both cytoplasm and the nucleus.

### Calmodulin specifically interacts with TBC1D3 in a Ca^2+^-dependent manner

**Figure 2:**
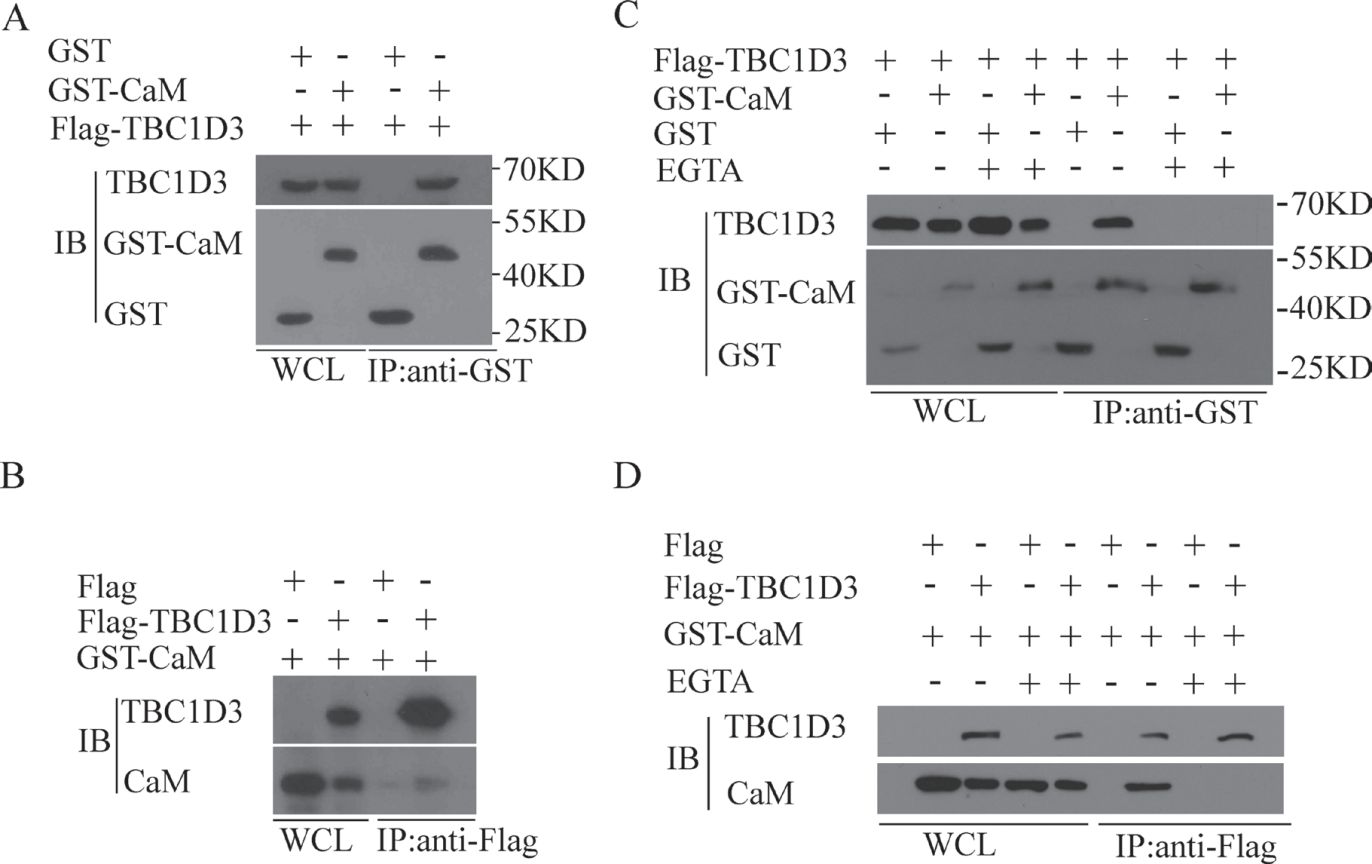
CaM associates with TBC1D3 in a Ca^2+^-dependent manner MCF-7 (**A**, **B** and **D**) and BT549 (**C**) cells were co-transfected with Flag-TBC1D3 together with GST-CaM or control GST vector (A and C), or with GST-CaM together with Flag-TBC1D3 or control Flag vector (B and D), and then treated with or without EGTA (2 mM). Lysates were immunoprecipitated (IP) with anti-GST (A and C) or anti-Flag (B and D) antibodies. After SDS-PAGE, Flag-TBC1D3 and GST-CaM/GST were immunoblotted (IB) with anti-Flag and anti-GST antibodies, respectively. Molecular weight markers are shown on right in kD. WCL, whole cell lysate.

We next sought to examine the mechanism by which CaM inhibits the GF-induced degradation of TBC1D3 in human breast cancer cells. CaM has been demonstrated to directly bind to TRE17/USP6, another TBC domain-containing oncogene that results from a chimeric fusion of the TBC1D3 and USP32 genes [39]. The N-terminal 499 amino acids of TRE17/USP6 exhibit 81% sequence identity with TBC1D3 [40]. These raised the possibility that CaM might inhibit the GF-induced ubiquitination and degradation of TBC1D3 through its interaction with TBC1D3. To test this, we performed the co-immunoprecipitation with anti-GST antibody. As seen in Figure 2A, TBC1D3 was not present in anti-GST immunoprecipitates from control GST vector-expressing cells. In contrast, TBC1D3 did exist in anti-GST immunoprecipitates from MCF-7 cells transiently transfected with GST-CaM (Figure 2A). Also, the reciprocal immunoprecipitation was performed in MCF-7 cells. As a negative control, CaM had no real existence in anti-Flag immunoprecipitates from control Flag vector-expressing cells (Figure 2B). In contrast, CaM did co-immunoprecipitate with Flag-TBC1D3 in the presence of anti-Flag antibody (Figure 2B). These data indicate that CaM specifically associates with TBC1D3.

Although CaM is an evolutionarily conserved Ca^2+^-binding protein that associates with its target proteins in a Ca^2+^-dependent manner, Ca^2+^-free CaM (apo-CaM) binds to other proteins independently of Ca^2+^ [18, 41]. Thus, we sought to determine whether CaM interaction with TBC1D3 was Ca^2+^-dependent. As seen in Figure 2C, TBC1D3 existed in anti-GST immunoprecipitates from BT549 cells transiently transfected with GST-CaM but not with control GST vector in the absence of EGTA, a Ca^2+^-chelating agent. However, addition of EGTA completely abolished the interaction between TBC1D3 and CaM (Figure 2C). Similarly, CaM was present and absent in anti-Flag immunoprecipitates from MCF-7 cells transiently transfected with Flag-TBC1D3 in the absence and presence of EGTA, respectively (Figure 2D). Collectively, these results reveal that CaM specifically interacts with TBC1D3 in a Ca^2+^-dependent manner.

### Mapping of the CaM-interacting site in TBC1D3

**Figure 3:**
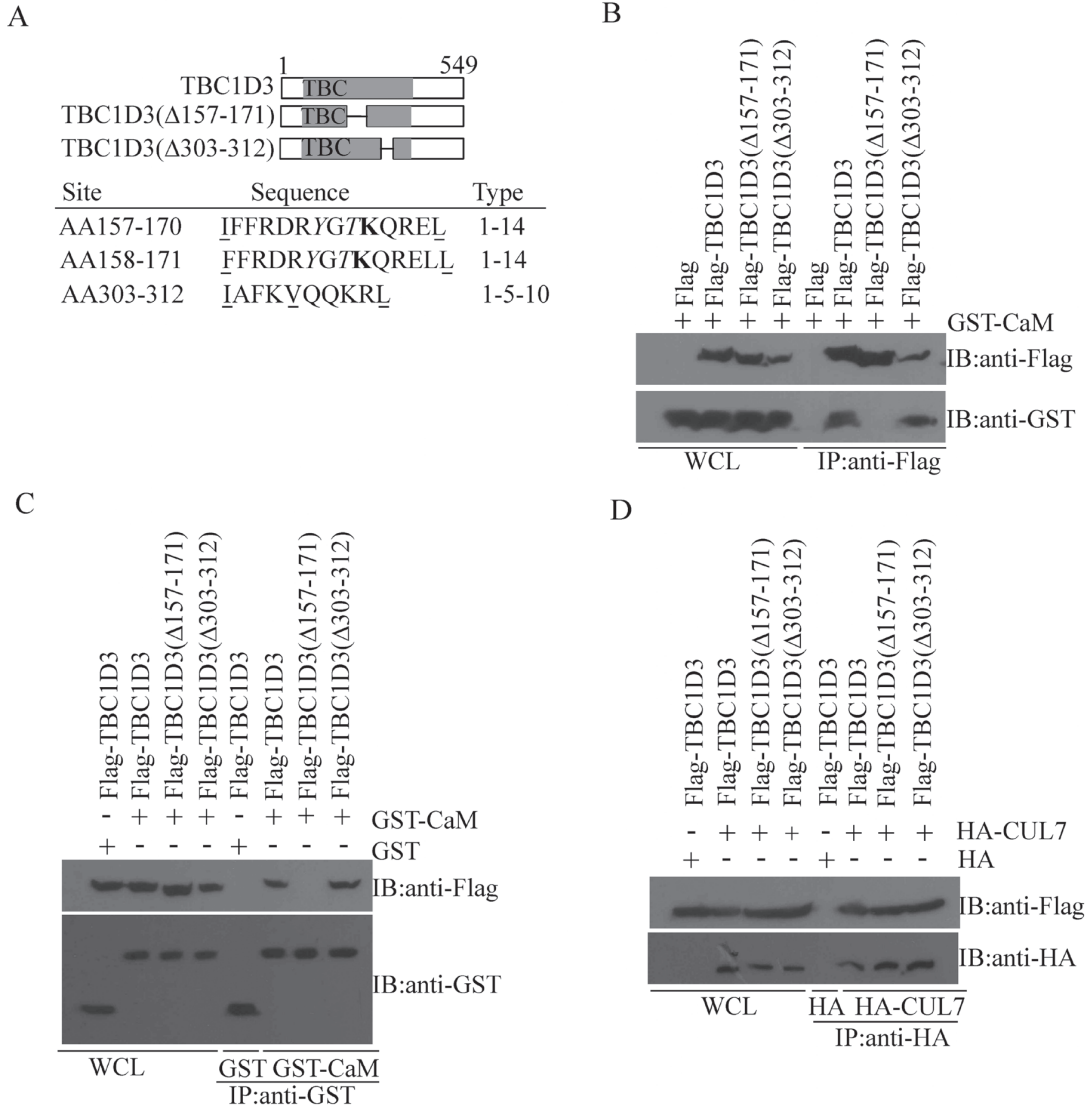
Mapping of the CaM-interacting site in TBC1D3 (**A**) Schematic representation of full-length TBC1D3 and various deletion mutants in top panel. The length or internal deletion (Δ) of each isoform in amino acids is indicated. TBC/Rab GAP homology domain is shown in gray. Three potential CaM-interacting motifs, two potential phosphorylatable amino acid residues and one potential ubiquitination site in TBC1D3 were identified in bottom panel. The position (in amino acids (AA)), sequence of the CaM-interacting sites (with conserved hydrophobic residues underlined, phosphorylatable residues italicized, and ubiquitination site boldfaced), and motif type are shown for each site. (**B**–**D**) MCF-7 cells were co-transfected with GST-CaM (B), GST-CaM/control GST (C), or HA-CUL7/control HA (D), together with control Flag vector, Flag-TBC1D3, or TBC1D3 mutants harboring internal deletions of the indicated residues. Lysates were immunoprecipitated (IP) with anti-Flag (B), anti-GST (C), or anti-HA (D) antibodies. After SDS-PAGE, Flag-TBC1D3, GST-CaM/GST, and HA-CUL7 were immunoblotted (IB) with anti-Flag, anti-GST and anti-HA antibodies, respectively. WCL, whole cell lysate.

CaM binds to and modulates an enormous variety of proteins, which usually possess the defined CaM-binding motifs, including IQ, 1-10, 1-14 and 1-16 motifs [18]. 1-10 and 1-14 motifs contain three (1-10, 1-5-10 and basic 1-5-10) and four subtypes (1-14, 1-8-14, basic 1-8-14 and 1-5-8-14), respectively, based on the indicated conserved hydrophobic amino acid positions. The CaM-binding motifs are often characterized by amphipathic helices, moderate hydrophilicity, net positive charge, and hydrophobic anchor residues [18]. Relying on these biochemical and biophysical characteristics, the web-accessible algorithm (http://calcium.uhnres.utoronto.ca/ctdb/ctdb/home.html, Calmodulin Target Database) predicted three putative CaM-interacting sites in TBC1D3, including two 1-14 and one 1-5-10 motifs (bottom panel in Figure 3A). To determine whether any of these motifs mediates TBC1D3 interaction with CaM, we constructed internal deletion mutants TBC1D3(Δ157-171) and TBC1D3(Δ303-312), which are deficient in the two 1-14 and one 1-5-10 motifs, respectively (Figure 3A), and then performed the co-immunoprecipitation with anti-Flag antibody. As seen in Figure 3B, CaM associated strongly with wild-type TBC1D3 and the 1-5-10 motif-deficient mutant TBC1D3(Δ303-312), but not with control Flag vector in the presence of anti-Flag antibody. In contrast, deficiency in the two 1-14 motifs (i.e. amino acids 157-171) abolished interaction between TBC1D3 and CaM (Figure 3B). Consistent with these results, both TBC1D3 and TBC1D3(Δ303-312) were present in anti-GST immunoprecipitates from MCF-7 cells transiently transfected with GST-CaM but not with control GST vector, whereas the two 1-14 motifs-deficient mutant TBC1D3(Δ157-171) failed to associate with CaM (Figure 3C).

To ensure that the CaM interacting-deficient mutant TBC1D3(Δ157-171) was well folded and functional, we confirmed its ability to associate with known binding partners, such as CUL7 [34], as efficiently as wild-type TBC1D3 and the 1-5-10 motif-deficient mutant TBC1D3(Δ303-312). In contrast, as a negative control, wild-type TBC1D3 disappeared in anti-HA immunoprecipitates from MCF-7 cells transiently transfected with control HA vector (Figure 3D). In aggregate, these results indicate that the CaM-interacting site in TBC1D3 resides at or near amino acids 157–171, which comprises two 1-14 motifs (Figure 3A).

### Point mutation of the ubiquitination site K166 abolishes the FCS-induced ubiquitination and degradation of TBC1D3

**Figure 4:**
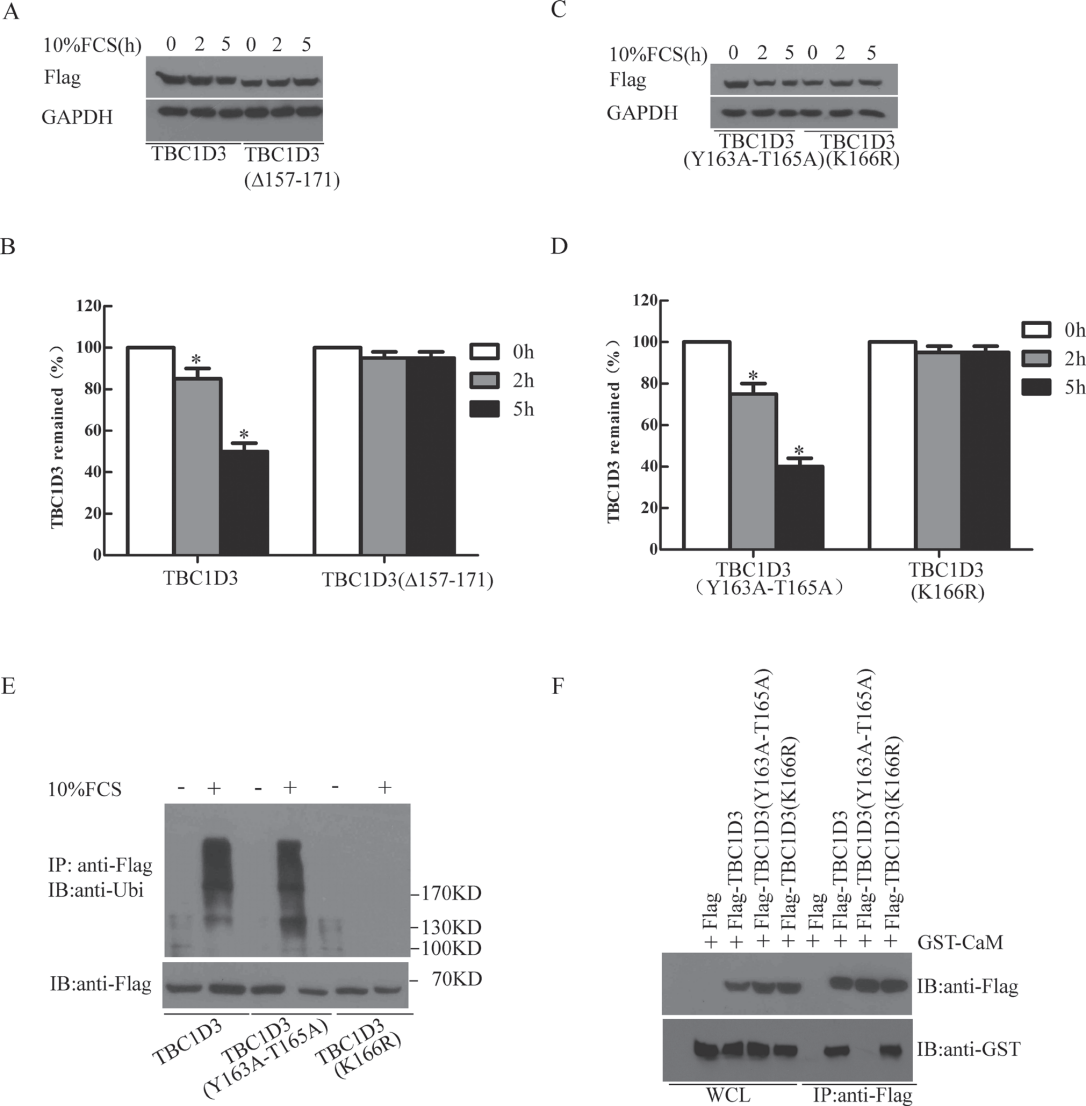
Mutation of the ubiquitination site K166 abolishes the ubiquitination and degradation of TBC1D3 in response to FCS stimulation (**A**–**D**) MCF-7 cells were transfected with Flag-tagged wild-type (A, B), internal deletion mutant (A, B), or point mutants (C, D) of TBC1D3. After 20 h, the cells were starved in serum-free medium for 3 h, and then stimulated with or without 10% fetal calf serum (FCS) in the presence of cycloheximide (25 μg/ml) for the indicated times. Cell extracts were resolved by SDS-PAGE and immunoblotted with anti-Flag and anti-GAPDH antibodies. The bar graphs B and D are derived from densitometric analysis of Western blots as typified in A and C, respectively. Expression of TBC1D3 and its mutant is normalized to GAPDH. The initial level of TBC1D3 expression in each group is set to 100%. The data are presented as means ± SD of three independent experiments (*p < 0.05). (**E**) MCF-7 cells were transfected with Flag-tagged wild-type or indicated point mutants of TBC1D3. After 20 h, the cells were serum-starved in the presence of MG132 for 6 h, and then stimulated with (+) or without (−) 10% FCS for 20 min. Lysates were immunoprecipitated (IP) with anti-Flag antibody. After SDS-PAGE, non- and poly-ubiquitinated forms of Flag-TBC1D3 and its point mutants were immunoblotted (IB) with anti-Flag and anti-ubiquitin (Ubi) antibodies, respectively. Molecular weight markers are shown on right in kD. (**F**) MCF-7 cells were co-transfected with GST-CaM together with control Flag vector, Flag-tagged wild-type or indicated point mutant of TBC1D3. Lysates were immunoprecipitated (IP) with anti-Flag antibody. After SDS-PAGE, Flag-tagged wild-type or indicated point mutants of TBC1D3 and GST-CaM were immunoblotted (IB) with anti-Flag and anti-GST antibodies, respectively. WCL, whole cell lysate.

Since CaM specifically interacts with TBC1D3 and inhibits its degradation in response to GF signaling, we anticipate that the deficiency in CaM interacting would promote the degradation of TBC1D3. However, immunoblotting revealed that deletion mutations of the two 1-14 motifs (i.e. amino acids 157–171) caused inability of FCS stimulation to induce the degradation of TBC1D3 (Figure 4A and 4B). Given this discrepancy, we inspected the amino acid composition of TBC1D3 protein sequences in greater detail. Amino acids 157–171 within TBC1D3 contain two phosphorylatable amino acid residues (Y163 and T165) and one lysine residue (K166) (bottom panel in Figure 3A), which might be modified by a variety of PTMs including ubiquitination [42]. Since the phosphorylation and subsequent polyubiquitination of TBC1D3 is required for the FCS-induced degradation of the oncoprotein [34], this raised two possibilities. One was that amino acid residues Y163 or/and T165 might be a phosphorylation site required for TBC1D3 association with F-box 8 (Fbxw8), the substrate recognition domain of SCF-FBXW8 E3 ubiquitin ligase, and for its subsequent degradation. Another possibility, which is not mutually exclusive, was that amino acid residue K166 might be an ubiquitination site necessary for TBC1D3 ubiquitination and subsequent degradation. To explore these possibilities, point mutants TBC1D3(Y163A-T165A) and TBC1D3(K166R) were generated and expressed in MCF-7 cells. Similar to the CaM interacting-deficient mutant TBC1D3(Δ157-171) (Figure 4A and 4B), point mutation of the amino acid residue K166, but not Y163 and T165, abolished the FCS-induced degradation of TBC1D3 (Figure 4C and 4D), suggesting that CaM inhibits GF signaling-induced degradation of TBC1D3 through occluding its ubiquitination at K166. Supporting this notion, we detected a population of polyubiquitinated TBC1D3 in anti-Flag immunoprecipitates from MCF-7 cells transiently transfected with wild-type TBC1D3 or the phosphorylatable residue mutant TBC1D3(Y163A-T165A), but not with the ubiquitination site mutant TBC1D3(K166R) (Figure 4E). Notably, although mutation of the ubiquitination site had no effect on the interaction between TBC1D3 and CaM, mutation of the phosphorylatable residues Y163 and T165 did abolish such an interaction (Figure 4F), and caused an increased degradation of TBC1D3 (Figure 4A–4D), further suggesting that endogenous CaM is of great importance in the stability of TBC1D3. Taken together, these results suggest that CaM interacts with TBC1D3 and inhibits GF signaling-induced ubiquitination at K166 and subsequent degradation of the oncoprotein in human breast cancer cells.

### TBC1D3 promotes the migration of human breast cancer cells in a manner involving the expression and activation of MMP-9

**Figure 5:**
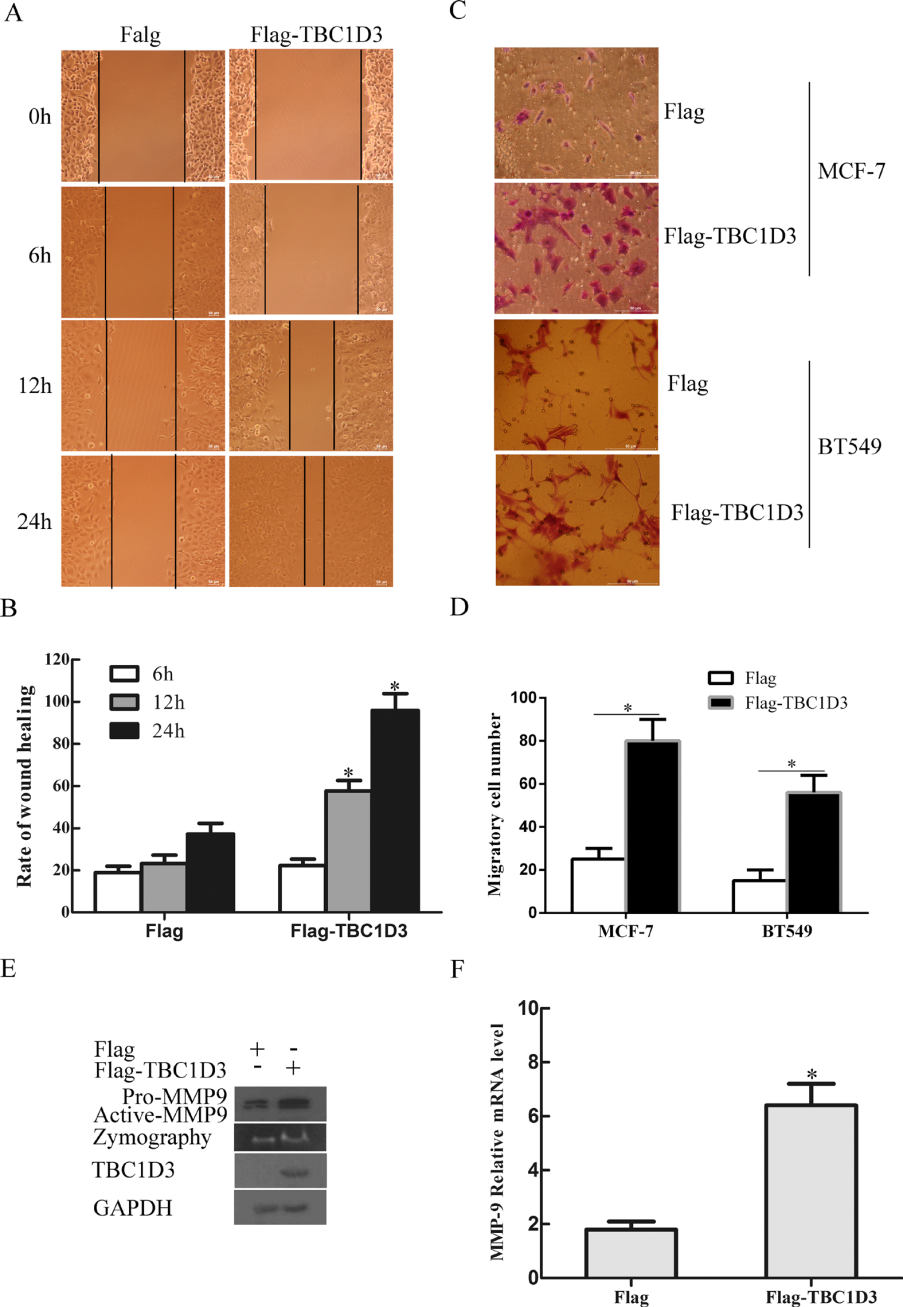
TBC1D3 overexpression promotes the expression and activation of MMP-9 and the migration of MCF-7 cells (**A**) MCF-7 cells were transfected with Flag-TBC1D3 or control Flag vector. After 20 h, the confluent monolayer of the transfected cells were scratched and then allowed to migrate into the wound area for the indicated times. Photographs were taken at a magnification of × 200. Scale bar, 50 μm. (**B**) The bar graph is derived from scratch wound assay as typified in A. The rate of cell migration was analyzed based on the percentage of the distance of cell migration into the wound area at the indicated time points over the initial wound width in each group, using ImageJ software. The data are presented as means ± SD of three independent experiments (*p < 0.05). (**C**) MCF-7 and BT549 cells were transfected with Flag-TBC1D3 or control Flag vector. After 20 h, the transfected cells were added into the upper chambers with non-coated membrane (8-μm and 12-μm pore size for MCF-7 and BT549 cells, respectively) and complete medium containing 20% FCS into the lower chambers of transwells. Following incubation for 24 h, cells migrating from the top side to the bottom side of the membrane were fixed, stained, and then photographed at a magnification of X 200. Scale bar, 50 μm. (**D**) The bar graph is derived from transwell cell migration assay as typified in C. Five randomly selected fields of cells were photographed and counted using ImageJ software. The data are presented as means ± SD of three independent experiments (*p < 0.05). (**E**) MCF-7 cells were transfected with Flag-TBC1D3 or control Flag vector, and then starved in serum-free medium for 24 h. Whole cell lysates were immunoblotted for MMP-9 (including pro-MMP-9 and active MMP-9), Flag-TBC1D3, and GAPDH as a loading control. MMP-9 activity in concentrated conditioned medium was detected by gelatin zymography. (**F**) MCF-7 cells were transfected with Flag-TBC1D3 or control Flag vector, and then starved in serum-free medium for 24 h. Total RNA was extracted from the transfected cells and reverse-transcribed to cDNA. Subsequently, expression of MMP-9 mRNA in each group was analyzed by real-time quantitative PCR and normalized to GAPDH. The data are presented as means ± SD of three independent experiments (*p < 0.05).

Recently, TBC1D3 was shown to enhance GF signaling [31], which plays a critical role in metastasis of breast cancer cells to other organs [43]. However, it was unknown whether TBC1D3 had any effect on the migration and metastasis of breast cancers. To address these issues, we measured the migration of MCF-7 cells transfected with control Flag vector and Flag-TBC1D3 at different time points in a wound-healing assay. When compared with cells transfected with control Flag vector, TBC1D3-overexpressing cells exhibited an approximately 2.5 fold increase in motility at both 12 h and 24 h although there was no significant difference at 6 h (Figure 5A and 5B). Consistent with these results, the transwell cell migration assay, an alternative approach to study cell motility, revealed that MCF-7 and BT549 cells transfected with Flag-TBC1D3 had an increased migratory potential when compared with control Flag vector (Figure 5C and 5D). These results indicate that TBC1D3 overexpression promotes the migration of human breast cancer cells.

GF signaling has been shown to stimulate the expression of matrix metalloproteinases-9 (MMP-9), one of human zinc-binding endopeptidases, in breast cancer cells [44]. Since MMP-9 can promote the migration and metastasis of tumor cells through degradation of extracellular matrix (ECM) components [45], we next examined the effect of TBC1D3 on the expression and activation of this endopeptidase in MCF-7 cells. As seen in Figure 5E, the level of both pro-MMP-9 and active-MMP-9 proteins was significantly increased in MCF-7 cells transfected with TBC1D3 when compared with control Flag vector. These TBC1D3-overexpressing cells also displayed the increased activity of MMP-9, as monitored by gelatin zymography (Figure 5E). Consistent with these results, overexpression of TBC1D3 stimulated the up-regulation of MMP-9 mRNA in MCF-7 cells (Figure 5F), suggesting that TBC1D3 promotes the production of MMP-9 at a transcriptional level. Together, these data indicate that TBC1D3 promotes the migration of human breast cancer cells in a manner involving the expression and activation of MMP-9.

### CaM enhances the TBC1D3-induced expression and activation of MMP-9 and migration of human breast cancer cells

**Figure 6:**
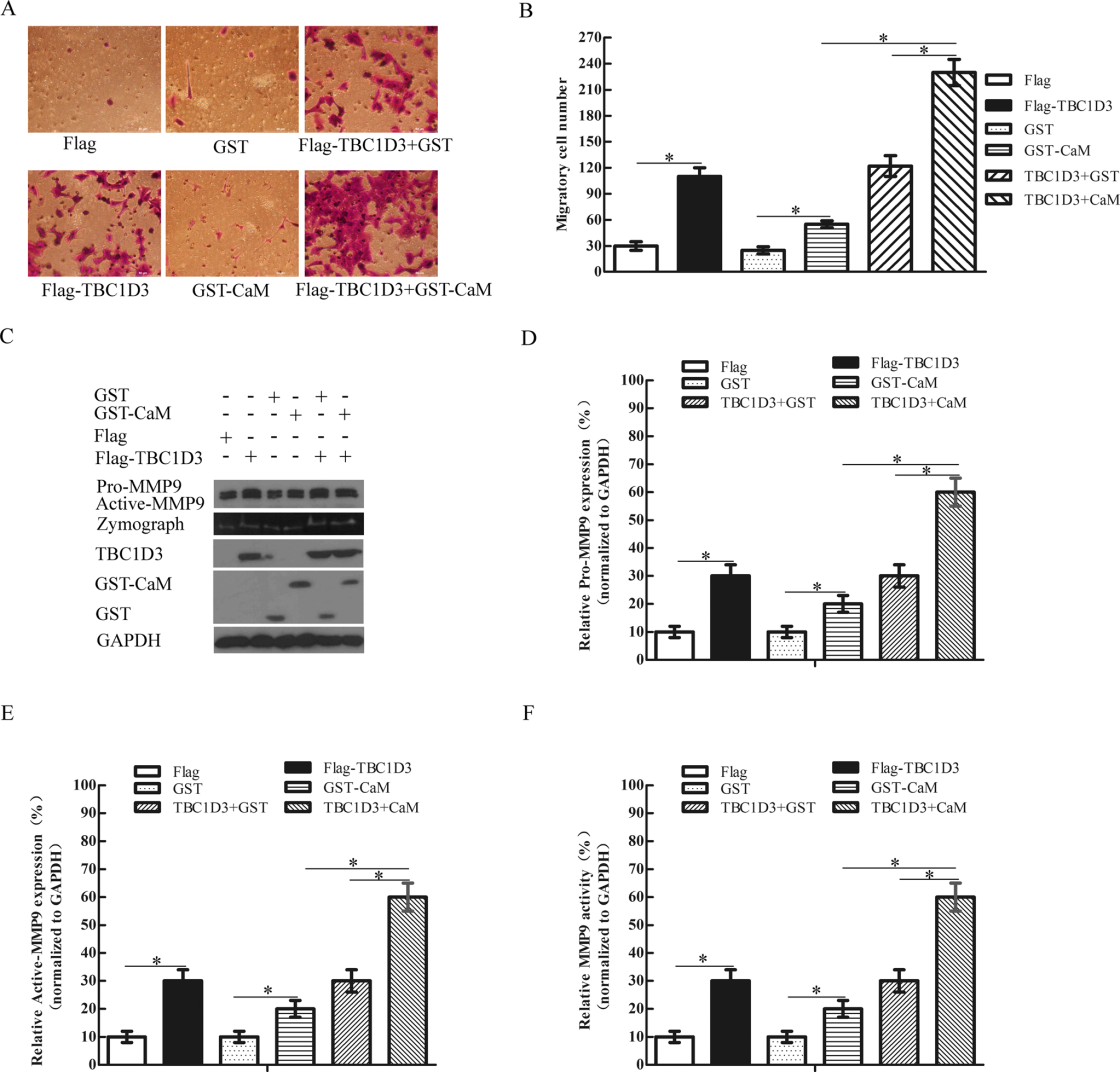
CaM enhances the TBC1D3-induced expression and activation of MMP-9 and migration of MCF-7 cells (**A**) MCF-7 cells were transfected with the indicated plasmids. After 20 h, the transfected cells were added into the upper chambers with non-coated membrane (8-μm pore size) and complete medium containing 20% FCS into the lower chambers of transwells. Following incubation for 24 h, cells migrating from the top side to the bottom side of the membrane were fixed, stained, and then photographed at a magnification of X 200. Scale bar, 50 μm. (**B**) The bar graph is derived from transwell cell migration assay as typified in A. Five randomly selected fields of cells were photographed and counted using ImageJ software. The data are presented as means ± SD of three independent experiments (*p < 0.05). (**C**) MCF-7 cells were transfected with the indicated plasmids, and then starved in serum-free medium for 24 h. Whole cell extracts were immunoblotted for MMP-9 (including pro-MMP-9 and active MMP-9), Flag-TBC1D3, GST, GST-CaM and GAPDH. MMP-9 activity in concentrated conditioned medium was detected by gelatin zymography. (**D**–**F**) The bar graphs D, E and F are derived from densitometric analysis of Western blots as typified in C. Expression Pro-MMP-9 (D) and active MMP-9 (E) as well as MMP-9 activity (F) is normalized to GAPDH. The data are presented as means ± SD of three independent experiments (*p < 0.05).

Since CaM inhibits the FCS-induced ubiquitination and degradation of TBC1D3 (Figure 1A–1D), we examined whether CaM affected the TBC1D3-induced migration of MCF-7 cells. When compared with control vector, transfection with Flag-TBC1D3 alone or together with control GST vector caused a robust increase in migratory potential of MCF-7 cells while expression of CaM alone only slightly increased the migration of these cells (Figure 6A and 6B). However, CaM coexpression considerably enhanced such effect of TBC1D3 (Figure 6A and 6B). Together, these data indicate that CaM expression enhances the TBC1D3-induced migration of human breast cancer cells.

We next examined the impact of CaM on the TBC1D3-induced expression and activation of MMP-9 in MCF-7 cells. Consistent with the results shown in Figure 5E, TBC1D3 overexpression caused the elevated expression and activation of MMP-9 in MCF-7 cells when compared with control Flag vector (Figure 6C–6F). Notably, coexpression of CaM, but not control GST, substantially enhanced such effect of TBC1D3 while transfection of MCF-7 cells with CaM alone only slightly increased the expression and activity of MMP-9 protein (Figure 6C–6F). Taken together, these data indicate that CaM enhances the TBC1D3-induced migration of human breast cancer cells by a mechanism involving the expression and activation of MMP-9.

## DISCUSSION

Our studies identify CaM as a novel regulator of TBC1D3. CaM specifically interacts with TBC1D3 in a Ca^2+^-dependent manner and inhibits GF signaling-induced ubiquitination and degradation of the oncoprotein in both cytoplasm and the nucleus of human breast cancer cells. Interaction of CaM is mediated by amino acids 157–171 within TBC1D3, which comprises two 1–14 motifs and one lysine residue (K166). Deletion mutation in these two 1-14 motifs is shown to abolish the interaction of TBC1D3 with CaM, and surprisingly, this mutation causes inability of GF signaling to induce the ubiquitination and subsequent degradation of TBC1D3. In agreement with this, point mutation of the lysine residue K166 exhibits the same effect on TBC1D3. Notably, our results indicate that TBC1D3 promotes the expression and activation of MMP-9 and the migration of MCF-7 cells. Furthermore, we show that interaction with CaM considerably enhances such effect of TBC1D3.

Our deletion mutation studies indicate that amino acids 157–171 within TBC1D3 are required for CaM to inhibit GF signaling-induced degradation of this oncoprotein. This mutation site contains two phosphorylatable amino acid residues (Y163 and T165) and one lysine residue (K166), which might be modified by a variety of PTMs including ubiquitination [42]. Therefore, two possible mechanisms by which CaM could suppress GF signaling-induced degradation of TBC1D3 are by inhibiting the phosphorylation or ubiquitination of this mutation site. The phosphorylation of TBC1D3 is required for the recruitment of Fbxw8, the substrate recognition domain of SCF-FBXW8 E3 ubiquitin ligase, and for its subsequent ubiquitination and degradation in response to GF signaling [34]. However, our work reveals that point mutation of amino acid residues Y163 and T165 fails to abolish the FCS-induced degradation of TBC1D3, suggesting that the first mechanism is unlikely. Nevertheless, given the spatial conformations of proteins, it remains possible that CaM inhibits the phosphorylation of TBC1D3 at phosphorylatable amino acid residues beyond the above deletion mutation site, which are required for TBC1D3 binding to SCF-FBXW8 E3 ubiquitin ligase. To determine the phosphorylation site of TBC1D3 in response to GF signaling and to develop a convenient means to detect phosphorylated TBC1D3 will contribute to clarification of the function.

In this study, we identify lysine 166 as the actual site for GF signaling-induced ubiquitination of TBC1D3. Point mutation of this lysine residue causes inability of GF signaling to induce the ubiquitination and subsequent degradation of TBC1D3. Notably, this ubiquitination site resides within the CaM-interacting motifs of TBC1D3. Therefore, it seems highly likely that CaM inhibits GF signaling-induced degradation of TBC1D3 by occluding its ubiquitination at K166. Consistent with this notion, CaM has been shown to suppress the degradation of several CaM-binding proteins, such as neuronal nitric-oxide synthase (nNOS) and estrogen receptor-α (ER-α), by a similar mechanism involving ubiquitination sites [46–49]. The ubiquitination sites of ER-α and nNOS, like that of TBC1D3, exist within their CaM-binding motifs, suggesting that the ubiquitination site appears to co-cluster with CaM-binding motif. In the presence of molecular chaperone heat shock protein 90 (Hsp90), CaM binding reduces the ubiquitination and degradation of nNOS although CaM alone does not have such effect [47]. Similarly, CaM uses its exposed glutamate residues (E11, E14, E84 and E87) to form salt bridges with key lysine residues (K299, K302 and K303) of the ubiquitination sites in ER-α, thereby attenuating the interaction of E3 ubiquitin ligase E6-associated protein (E6AP) to inhibit the ubiquitination at these sites and subsequent degradation of ER-α [48, 50]. Point mutation of lysine 299 in ER-α to alanine weakens its binding to CaM [48]. Unlike ER-α, we observed that substitution of lysine 166 in TBC1D3 by arginine, a basic amino acid like lysine, did not alter its interaction with CaM, indicating that the ubiquitination site is not essential for TBC1D3 interaction with CaM. Whether CaM regulates TBC1D3 functions not involving ubiquitination and degradation remains to be determined.

Our study identifies TBC1D3 as a novel stimulator of cell migration, and reveals that MMP-9 is involved in this migratory process. Multiple regulatory modes of MMP-9 have emerged, including the regulation at the transcriptional level and through the post-transcriptional events: secretion, activation, endogenous inhibitors and cell surface interaction [51]. Our real-time quantitative reverse transcription PCR (RT–qPCR) study shows the increased level of MMP-9 mRNA in MCF-7 cells transfected with TBC1D3, indicating that TBC1D3 increases MMP-9 expression at the transcriptional level. However, it remains to be determined how TBC1D3 stimulates its transcription. Recently, TBC1D3 was showed to enhance GF signaling [31], which has also been shown to stimulate the expression of MMP-9 at the transcriptional level in breast cancer cells [44]. Thus, it is more likely that TBC1D3 promotes the transcription of MMP-9 in a manner involving GF signaling pathway. In addition, we observed that TBC1D3 overexpression had no impact on the morphological features and the protein level of E-cadherin, an EMT marker, in MCF-7 cells (Supplementary Figures 2 and 3). However, we cannot exclude the possibility that EMT is also involved in the TBC1D3-induced cell migration, because there is evidence that loss of E-cadherin is not a necessity for epithelial to mesenchymal transition in human breast cancer [52].

The list of CaM-interacting proteins that regulate cell migration has grown rapidly in recent years, and multiple mechanisms by which CaM modulates cell migration through interaction with these proteins have emerged. The earliest identified mechanism involved Ca^2+^/CaM kinase II (CaMK II), which is activated by binding Ca^2+^-bound CaM [53]. Activation of CaMK II stimulates cell migration through a variety of pathways [54–61]. Other mechanisms by which CaM regulates cell migration and invasion are by interaction with various CaM-binding proteins such as Grb7, Na^+^/H^+^ exchanger NHE1 and HRPAP20 (hormone-regulated proliferation-associated protein 20) [62–64]. To our knowledge, however, CaM does not affect the ubiquitination and degradation of the CaM-binding proteins above mentioned. Our current study indicates that TBC1D3 serves as an additional CaM-interacting protein to stimulate cell migration, and proposes a novel mode by which CaM promotes cell migration through inhibiting the ubiquitination and degradation of TBC1D3.

## MATERIALS AND METHODS

### Plasmids

Flag-CUL7/pcDNA3.0 was generously provided by Dr. Zhen-Qiang Pan (Dept. of Oncological Science, Mount Sinai School of Medicine). HA-CUL7/pcDNA3.0 was obtained from AddGene. Hemagglutinin (HA)-TBC1D3/pcDNA3.0, pEGFP-TBC1D3 and CaM/pEBG have been described previously [33, 39]. The *Bam*H I*/Xho* I fragment encompassing the entire open reading frame (ORF) of TBC1D3 was subcloned into a modified version of pcDNA3.0 containing a Flag epitope tag (Flag-pcDNA3.0) to generate Flag-tagged fusion proteins (Flag-TBC1D3). Flag-TBC1D3(Δ157-171)/pcDNA3.0 and Flag-TBC1D3(Δ303-312)/pcDNA3.0, harboring internal deletions of the indicated residues, were generated by overlap extension PCR. Point mutants Flag-TBC1D3(Y163A-T165A)/pcDNA3.0 and Flag-TBC1D3(K166R)/pcDNA3.0 were also generated by overlap extension PCR. Further details are available upon request.

### Antibodies

For immunoprecipitation, antibodies against Flag (sc-166355) and HA (sc-805) were purchased from Santa Cruz. Anti-GST antibody (#2624, Cell Signaling Technology) was used for both immunoprecipitation and immunofluorescence (1:800). For immunoblotting, antibodies against Flag (sc-807, 1:10000; sc-166355, 1:15000), ubiquitin (sc-8017, 1:2000), and PARP-1 (sc-8007, 1:2000) were purchased from Santa Cruz, antibodies against MMP-9 (#10375-2-AP, 1:2000) and E-cadherin (#20874-1-AP, 1:2000) from Proteintech Group, antibodies against HA (#2367, 1:2500) and GAPDH (AP0063, 1:150000) were from Cell Signaling Technology and Bioworld Technology, respectively. The secondary antibody used for immunofluorescence was Rhodamine (TRITC)-conjugated goat anti-mouse immunoglobulin G (1:50, Jackson ImmunoResearch Laboratories).

### Cell culture and transfection

Human MCF-7 (ATCC) and BT549 (ATCC) breast carcinoma cells were grown in 5% CO_2_ at 37°C in Dulbecco′s Modified Eagle′s Medium (DMEM, Invitrogen) and Roswell Park Memorial Institute (RPMI) 1640 medium (Invitrogen), respectively, which contained 2 mM of glutamine and supplemented with 10% heat-inactivated fetal bovine serum, 100 U/ml of penicillin and 100 μg/ml of streptomycin at 37°C in 5% CO_2_. Cells were transfected using SuperFectin^TM^ II (PuFei Biotech) Reagent according to manufacturer′s instructions. Approximately 20 h after transfection, cells were processed as described for each experiment.

### TBC1D3 degradation and Western blot assays

MCF-7 and BT549 cells were co-transfected with Flag-TBC1D3 together with GST-CaM or control GST vector. After 20 h, the cells were starved in serum-free medium for 3 h, and then stimulated with or without 10% fetal calf serum (FCS) in the presence of cycloheximide (25 μg/ml) for different time points. Subsequently, the cells were washed with ice-cold phosphate-buffered saline (PBS) and lysed in lysis buffer (50 mM Tris-HCl, pH 7.5, 100 mM NaCl, 1% Triton X-100, 10% glycerol, 1 mM phenylmethylsulfonyl fluoride (PMSF), 2 μg/ml aprotinin, 5 μg/ml leupeptin, 5 μg/ml pepstatin, 10 mM sodium fluoride, 1 mM sodium orthovanadate). The lysates were clarified by centrifugation, and the clarified supernatant were separated by SDS PAGE and electroblotted onto a 0.45-μm pore PVDF membrane, which was blocked in 5% non-fat milk in TBST buffer (10 mM Tris-HCl [pH 7.5], 100 mM NaCl, 0.05% Tween 20), probed by an overnight incubation with anti- Flag, anti-GST and anti-GAPDH antibodies, washed with TBST and incubated with horseradish peroxidase-conjugated goat anti-rabbit IgG (Santa Cruz Biotechnology) or goat anti-mouse IgG (Bioworld Technology) for detection by SuperSignal West Pico Chemiluminescent Substrate (Pierce). Immunoblotting data were quantified by Image-Pro Plus 6.0 software (Media Cybernetics).

### Co-immunoprecipitation

Cells were lysed in ice-cold lysis buffer (50 mM Tris-HCl, pH 7.5, 100 mM NaCl, 1% Triton X-100, 1 μM CaCl_2_, 10% glycerol, 5 mM β-glycerophosphate, 1 mM PMSF, 2 mg/ml aprotinin, 5 mg/ml leupeptin, 5 mg/ml pepstatin). The lysates were clarified by centrifugation at 4°C. An aliquot of the clarified supernatant was removed for direct immunoblotting, and the remainder was incubated with anti-Flag, anti-GST, or anti-HA antibodies overnight at 4°C and then with Protein G PLUS-Agarose beads (Santa Cruz Biotechnology) for an additional 4 h. The beads were washed three times in the lysis buffer. The immunoprecipitates were resolved by SDS PAGE and then analyzed by immunoblotting.

### Ubiquitination assay

MCF-7 and BT549 cells were transfected with Flag-TBC1D3 alone or together with GST-CaM. After 20 h, the cells were serum-starved in the presence of MG132 (20 μM) for 6 h, stimulated with 10% FCS for 20 min, and then lysed in lysis buffer (50 mM Tris-HCl, pH 7.5, 100 mM NaCl, 1% SDS, 1% Triton X-100, 10% glycerol, 10 mM *N-ethylmaleimide*, 10 mM sodium fluoride, 1 mM sodium orthovanadate, 1 mM PMSF, 2 μg/ml aprotinin, 5 μg/ml leupeptin and 5 μg/ml pepstatin). The lysates were sonicated four times at 4°C for 30 seconds with a 30 seconds cooling period between each burst and then clarified by centrifugation at 4°C. An aliquot of the clarified supernatant was removed for direct immunoblotting, and the remainder was boiled for 5 min, diluted in 10 volumes of the lysis buffer without SDS, and then subjected to immunoprecipitation and immunoblot analysis.

### Nuclear and cytoplasmic protein extraction

Cytoplasmic and nuclear proteins were extracted using the Nuclear and Cytoplasmic Protein Extraction Kit (Beyotime Biotechnology) according to manufacturer's instructions. Briefly, MCF-7 and BT549 cells were washed with ice-cold PBS, resuspended in hypotonic cytoplasmic extraction buffer A supplemented with 1 mM PMSF, and then incubated for 15 min on ice. Subsequently, cytoplasmic extraction buffer B was added, and the extracts were incubated for 1 min on ice. After centrifugation at 12,000 g for 5 min at 4°C, the supernatant (cytoplasmic fraction) was collected and the cell pellet (containing nuclei) was resuspended in ice-cold nuclear extraction buffer C supplemented with 1 mM PMSF. Following incubation for 30 min on ice, the extracts were centrifuged at 14,000 g for 10 min at 4°C. The supernatant was collected as nucleic fraction. The cytoplasmic and nucleic fractions were resolved by SDS PAGE and then immunoblotted with anti-Flag, anti-GST, anti-PARP-1 and anti-GAPDH antibodies.

### Scratch wound assay

Artificial wounds were made by scratching the monolayer of confluent MCF-7 cells with a 10-μl pipette tip. After the wounds were extensively washes twice with PBS to remove non-adherent cells, fresh serum-free DMEM medium was replaced, and cells were allowed to migrate into the wound area. Photographs were taken with PowerShot G10 camera (Canon) at the indicated time points. The rate of cell migration was measured using ImageJ software (NIH).

### Transwell cell migration assay

MCF-7 and BT549 cells were transfected with the indicated plasmids. After 20 h, the transfected cells (1 × 10^5^) were added into the upper chamber with non-coated membrane (8-μm and 12-μm pore size for MCF-7 and BT549, respectively) and 600 μl of complete medium containing 20% FCS into the lower chamber of 24-well transwell (Corning). After incubation at 37°C for 24 h, cells remaining on the top side of the membrane were wiped off with cotton swabs, and cells migrating to the bottom side of the membrane were fixed with 4% paraformaldehyde and stained with 0.5% crystal violet. Then five randomly selected fields of cells were photographed at a magnification of × 200 using a microscope and counted using ImageJ software. The data are presented as means ± SD of three independent experiments.

### Gelatin zymography

MCF-7 cells were starved in serum-free medium for 24 h. Subsequently, conditioned medium was collected and concentrated to one-tenth of its original volume using centrifugal filters (Millipore). The concentrated medium was resolved in non-reducing 8% SDS PAGE containing 0.1% of gelatin. After electrophoresis, the gel was washed in zymogram renaturing buffer (2.5% Triton X-100) for 60 minutes to remove SDS, and incubated in zymogram developing buffer (50 mM Tris-HCl, pH 8.0, 5 mM CaCl_2_, 0.2 M NaCl, and 1 μM ZnCl_2_) overnight at 37°C, to allow MMP digestion of its substrate gelatin. Then, the gels were stained with 0.25% Coomassie brilliant blue R250 for 2 h and destained with destaining solution (5% Methanol and 7% Acetic acid). MMP activities appeared as bright bands of digested gelatin against the Coomassie brilliant blue–stained dark blue background. MMP9 activity was identified based on gelatin lysis at molecular masses of about 82 kDa.

### Reverse transcription–quantitative PCR

Total RNA was extracted with RNAiso Plus (TaKaRa Biotechnology) from MCF-7 cells and reverse-transcribed to cDNA using PrimeScript^TM^ RT Master Mix (TaKaRa Biotechnology). Real-time quantitative PCR was then performed using SYBR^®^ Premix Ex Taq^™^ on an ABI Prism 7900 system (Applied Biosystems). Difference between gene expressions was presented as fold changes normalized to GAPDH level. Primers used to amplify MMP-9 in this experiment were as follows:

MMP-9 forward primer: 5′-TTGACAGCGACAAGA AGTGG-3′

MMP-9 reverse primer: 5′-GCCATTCACGTCG TCCTTAT-3′

### Statistical analysis

Data are expressed as the mean ± SD. The difference between the two groups was determined by Student's *t-test*, using SPSS software, version 18.0. A one-way analysis of variance (ANOVA) was used for multiple group comparisons. A *p-value* of < 0.05 was considered statistically significant.

## SUPPLEMENTARY MATERIALS FIGURES


