# Supplementary material for: Calmodulin promotes matrix metalloproteinase 9 production and cell migration by inhibiting the ubiquitination and degradation of TBC1D3 oncoprotein in human breast cancer cells
Source: Oncotarget. 2017 Mar 31;8(22):36383–98. doi: 10.18632/oncotarget.16756 (PMC5482662; doi:10.18632/oncotarget.16756)
Supplement: Supplementary file 1 [file oncotarget-08-36383-s001.pdf]

Calmodulin promotes matrix metalloproteinase 9 production and cell migration by inhibiting the ubiquitination and degradation of TBC1D3 oncoprotein in human breast cancer cells

Supplementary Materials

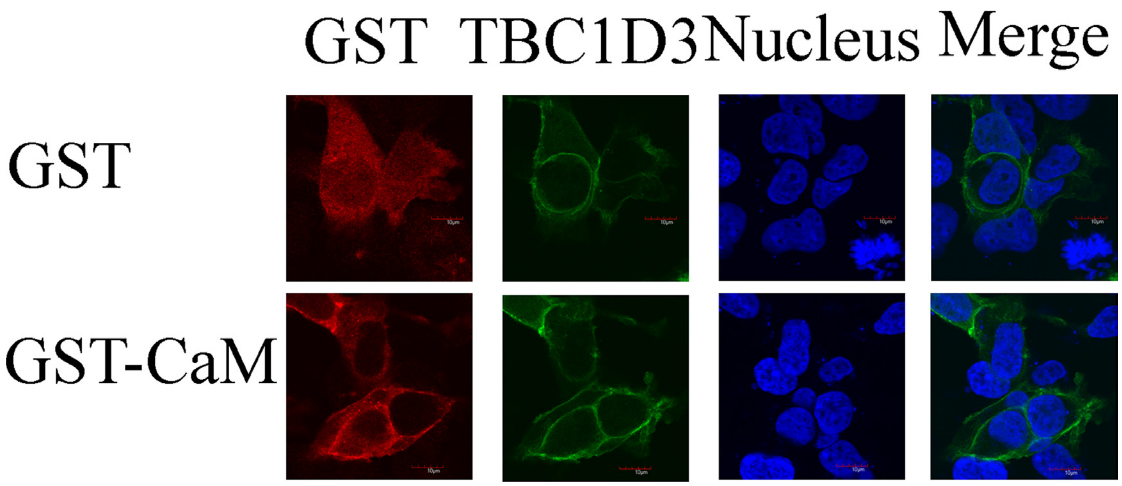

Supplementary Figure 1: CaM does not affect the TBC1D3 localization in MCF-7 cells. MCF-7 cells were co-transfected with EGFP-tagged TBC1D3 (green) together with control GST vector (top panels) or GST-CaM (bottom panels). Cells were subjected to indirect immunofluorescence with anti-GST (red), stained with Hoechst 33258 (blue), and then analyzed by confocal microscopy. Immunofluorescent images were merged with green and blue revealing TBC1D3 and nucleus, respectively (Merge). Scale bar, 10 µm.

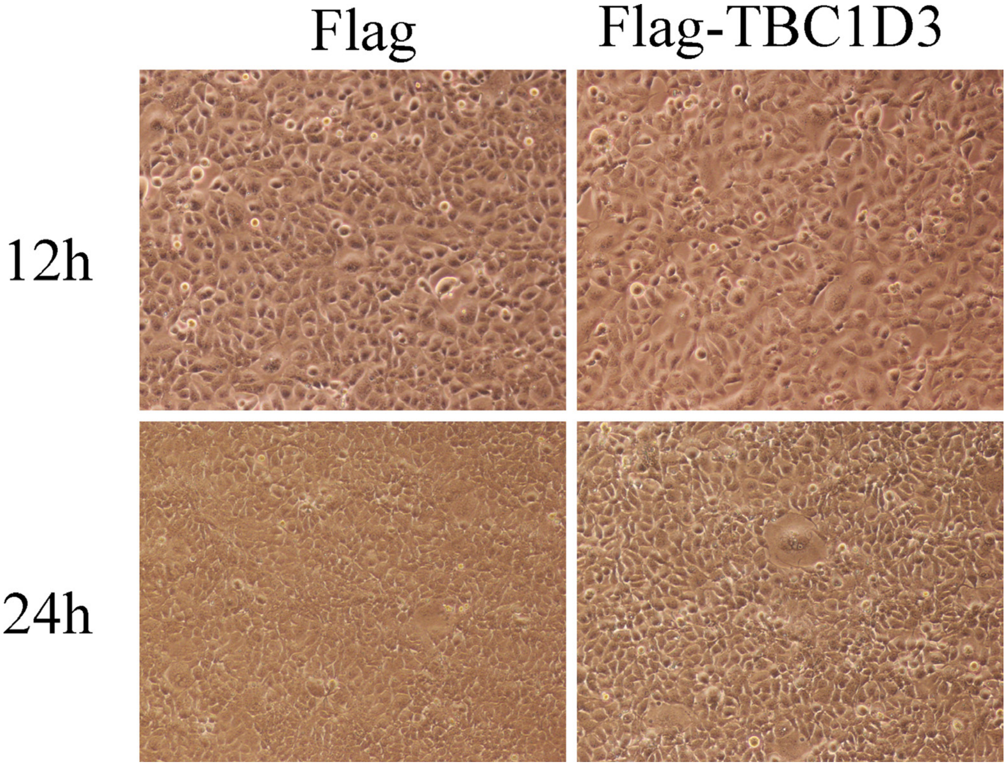

Supplementary Figure 2: TBC1D3 overexpression does not change the morphological features of MCF-7 cells. MCF-7 cells were transfected with Flag-TBC1D3 or control Flag vector. After 20 h, the transfected cells were resuspended in serum-free DMEM medium, and a suspension containing 1×10^5 cells was used for transwell cell migration assay. The rest of the suspension was seeded back into a 35 mm dish with 20% FCS and incubation at 37ºC for 24 h. Photographs were taken at the indicated time points.

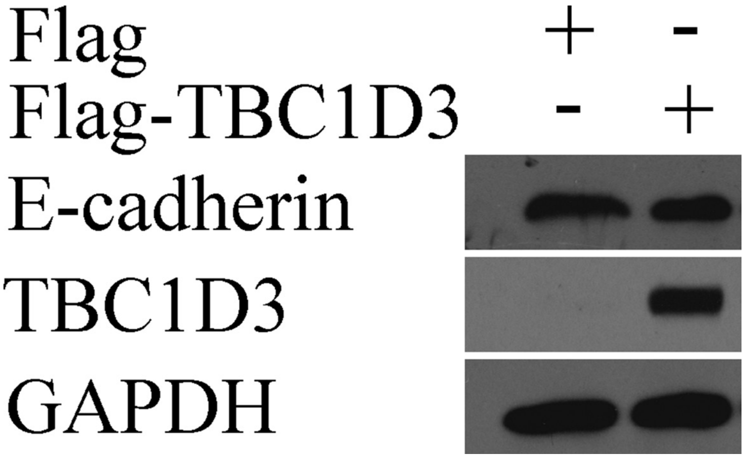

Supplementary Figure 3: TBC1D3 overexpression does not change the level of E-cadherin protein. MCF-7 cells were transfected with Flag-TBC1D3 or control Flag vector. After 20 h, cell extracts were resolved by SDS-PAGE and immunoblotted with anti-Flag (TBC1D3), anti-E-cadherin and anti-GAPDH antibodies.
